# Current Barriers to Clinical Liver Xenotransplantation

**DOI:** 10.3389/fimmu.2022.827535

**Published:** 2022-02-23

**Authors:** Arthur A. Cross-Najafi, Kevin Lopez, Abdulkadir Isidan, Yujin Park, Wenjun Zhang, Ping Li, Sezai Yilmaz, Sami Akbulut, Burcin Ekser

**Affiliations:** ^1^ Transplant Division, Department of Surgery, Indiana University School of Medicine, Indianapolis, IN, United States; ^2^ Department of Surgery and Liver Transplant Institute, Inonu University Faculty of Medicine, Malatya, Turkey

**Keywords:** liver xenotransplantation, thrombocytopenia, xenograft, immune rejection, porcine

## Abstract

Preclinical trials of pig-to-nonhuman primate liver xenotransplantation have recently achieved longer survival times. However, life-threatening thrombocytopenia and coagulation dysregulation continue to limit preclinical liver xenograft survival times to less than one month despite various genetic modifications in pigs and intensive pharmacological support. Transfusion of human coagulation factors and complex immunosuppressive regimens have resulted in substantial improvements in recipient survival. The fundamental biological mechanisms of thrombocytopenia and coagulation dysregulation remain incompletely understood. Current studies demonstrate that porcine von Willebrand Factor binds more tightly to human platelet GPIb receptors due to increased O-linked glycosylation, resulting in increased human platelet activation. Porcine liver sinusoidal endothelial cells and Kupffer cells phagocytose human platelets in an asialoglycoprotein receptor 1-dependent and CD40/CD154-dependent manner, respectively. Porcine Kupffer cells phagocytose human platelets *via* a species-incompatible SIRPα/CD47 axis. Key drivers of coagulation dysregulation include constitutive activation of the extrinsic clotting cascade due to failure of porcine tissue factor pathway inhibitor to repress recipient tissue factor. Additionally, porcine thrombomodulin fails to activate human protein C when bound by human thrombin, leading to a hypercoagulable state. Combined genetic modification of these key genes may mitigate liver xenotransplantation-induced thrombocytopenia and coagulation dysregulation, leading to greater recipient survival in pig-to-nonhuman primate liver xenotransplantation and, potentially, the first pig-to-human clinical trial.

## Introduction

End-stage organ failure currently plagues over 106,000 people in the United States (1). The only definitive treatment for this devastating condition is organ transplantation. Unfortunately, the demand for organs outpaces the supply, a scenario which has created a severe organ shortage. The inevitable reality is that many on the transplant waiting list will die before ever receiving a life-saving organ. In order to address these issues, the practice of xenotransplantation, i.e., transplanting organs from one species to another, was explored. Over the past few decades, the pig was identified as the ideal organ donor for transplantation into humans based on its current use as a source of food, rapid developmental timeline, relatively large litter size, roughly similar solid-organ size match, reasonable genetic similarity to humans, and its ability to be genetically-engineered (2). With the accessibility of genetically modified pigs, remarkable success has been achieved in pig-to-nonhuman primate (NHP) models. In a genetically modified pig-to-NHP model, life-supporting renal and heart xenografts have been reported to survive up to 435 days (3) and 195 days (4), respectively. A non-life-supporting pig-to-NHP heart xenograft survived for almost three years (945 days) (5).

Despite such promising advances in the field, the success of liver xenotransplantation has historically lagged behind its solid-organ counterparts. In contrast to the extended lifespan of heart and kidney xenografts, liver xenograft survival to date has been limited to less than one month (6, 7). Given the relatively shorter survival time of liver xenografts, the current potential clinical application of it is limited to a functional bridge to allotransplantation (8, 9). The primary barriers to successful liver xenotransplantation have consistently been the severe and rapid thrombocytopenia (10, 11) combined with uncontrollable coagulation dysregulation (11–13) that inevitably results in fatal hemorrhage of the recipient (9, 11, 13). The resolution of these major barriers may allow for the first pig-to-human liver xenotransplantation clinical trials as a bridge to allotransplantation, and potentially as definitive therapy for end-stage liver disease. We here provide a comprehensive review of the current status of pig-to-NHP liver xenotransplantation and the fundamental biological mechanisms of liver xenotransplantation-induced thrombocytopenia and coagulation dysregulation. Genetic modifications targeted at overcoming each of these obstacles will additionally be discussed.

## Clinical and Preclinical Experiences in Liver Xenotransplantation

### Wild-Type (WT) Pigs

Prior to produce of genetically-engineered pigs, (WT) pig livers were used in pig-to-NHP liver xenotransplantation. Survival times were limited, and recipient demise as a result of sequelae from hyperacute rejection was inevitable. If the recipient managed to evade the lethality of hyperacute rejection, coagulopathy followed by uncontrollable hemorrhage rapidly ensued (14). The first and only attempt at pig-to-human clinical liver xenotransplantation was performed by Makowka et al. (15) in 1995. The case has been described in extensive detail elsewhere (13, 15); however, it is of historical significance that this first attempt was made with a liver from a WT pig (15).

### Genetically Modified Pigs

The advent and widespread use of gene-editing tools renewed interest in liver xenotransplantation. Initial genetically engineered pig-to-NHP models targeted the complement cascade, in particular the expression of humanized complement regulatory proteins such as CD55 (also known as decay-accelerating factor, or DAF) in pigs (16, 17). By modulating the complement cascade, hyperacute antibody-mediated rejection could theoretically be ameliorated. In combination with immunosuppressive therapy, two baboon recipients survived for more than four days, surpassing all previous attempts with WT pig livers (16). The ability to genetically optimize pig organs to improve recipient survival was no longer a dream: it was the future of liver xenotransplantation.

Since the initial successful liver xenotransplantation preclinical trials using genetically modified pigs, there has been an explosion in the number of genetically modified pigs available for use in xenotransplantation research (18). Genetic modifications targeted at xenoantigens, complement pathways, and components of the coagulation cascade have been performed in order to increase graft survival times and have been tested in pig-to-NHP models (19, 20) (**Table 1**). Prior to 2016, survival times in preclinical liver xenotransplantation were limited to just over two weeks (**Table 1**). In 2016, Shah et al. (6) broke through this plateau by performing an orthotopic pig-to-baboon liver xenotransplantation in which the recipient survived a total of 25 days (6, 22). Key differences from prior attempts included the continuous infusion of human prothrombin complex concentrate (hPCC) aimed at mitigating coagulopathy as well as the addition of belatacept, a monoclonal antibody (mAb) directed against CD80/86, a receptor involved in T-cell costimulation (6, 22). A year later, the same group replaced belatacept with an anti-CD40 mAb as the primary agent for costimulation blockade, with all other parameters held constant. The result was an astounding survival time of 29 days in one of the recipients (7). The livers in these trials were from α-galactosyltransferase gene-knockout (GTKO) pigs with no additional genetic modifications. Combining multiple genetic modifications with promising new pharmacological strategies could potentially pave the way to prolonged recipient survival.

**Table 1:** Genetically-engineered pig-to-nonhuman primate liver xenotransplantation.

Year	Genetic Modifications	Recipient	Transplant	N	Pharmacologic Regimen	Survival	Reference
2000	hCD55	Baboon	Orthotopic	2	CyP CsA Cs	96, 192 h	(16)
2005	hCD55.CD50.HT	Baboon	Orthotopic	5	CyP CsA Cs Daclizumab Rituximab MMF	13, 18, 20, 21, 24 h	(17)
2010	GTKO	Baboon	Orthotopic	2	Cs MMF ATG Tacrolimus	3, 144 h	(10)
2010	GTKO.hCD46	Baboon	Orthotopic	5	Cs MMF ATG Tacrolimus	3, 20, 24, 96, 120 h	(10)
2010	GTKO.hCD46	Baboon	Orthotopic	3	CyP Cs MMF Tacrolimus	144, 144, 168 h	(10)
2012	GTKO	Baboon	Orthotopic	2	Cs ATG Tacrolimus CVF AZA anti-CD154 LoCD2b (one case)	144, 216 h	(19)
2012	MGH MS, GTKO	Baboon	Orthotopic	3	Cs ATG Tacrolimus CVF AZA anti-CD154 LoCD2b	6, 8, 9 days	(19)
2014	MGH MS, GTKO	Baboon	Heterotopic	3	Cs ATG Tacrolimus CVF	6, 9, 15 days	(20)
2015	WZ MS, GTKO	Tibetan monkey	Heterotopic	3	Cs MMF ATG Tacrolimus CVF anti-CD154 salviae miltiorrhizae	2, 5, 14 days	(21)
2016	MGH MS, GTKO	Baboon	Orthotopic	6	Cs ATG Tacrolimus CVF hPCC	1, 3, 4, 4, 6, 7 days	(22)
2016	MGH MS, GTKO	Baboon	Orthotopic	1	Cs ATG Tacrolimus CVF hPCC Belatacept	25 days	(6)
2017	MGH MS, GTKO	Baboon	Orthotopic	4	Cs ATG Tacrolimus CVF hPCC anti-CD40mAb	25, 5, 8, 29 days	(7)
2020	GTKO, CMAH-KO, B4GALNT2-KO, PERV-KO, hCD46, hCD55, hCD59, hTHBD, hTFPI, hCD39, hB2M, HLA-E, hCD47-TG	Rhesus monkey	Heterotopic	1	ATG anti-CD40mAb	26 days	Dou, personal communication

ATG, anti-thymocyte globulin; AZA, azathioprine; B4GALNT2, Beta-1,4-N-Acetyl Galactosaminyltransferase 2; CMAH, Cytidine monophospho-N-acetylneuraminic acid hydroxylase; Cs, corticosteroids; CsA, cyclosporine; Cyp, cyclophosphamide; GTKO, galactosyltransferase knockout; h, humanized; hPCC, human prothrombin complex concentrate; LoCD2b, rat anti-primate CD2 IgG2b; MGH MS, Massachusetts General Hospital miniature swine; MMF, mycophenolate mofetil; PERV, porcine endogenous retrovirus; WZ MS, Wu Zhanshen miniature swine.

In 2020, Dou et al. performed a ‘heterotopic’ pig-to-monkey liver xenotransplantation with splenectomy to achieve a 26-day of survive periods (personal communication by Dr. Kefeng Dou at the 2021 IXA meeting). The organ source pig was genetically modified to lack expression of key xenoantigens and to express complement regulatory proteins, coagulation cascade proteins, as well as several other genes (**Table 1**). In addition, certain modifications to the engraftment procedure were performed, including transection of the recipient inferior vena cava with subsequent end-to-end anastomoses from the donor portal vein to the distal inferior vena cava, donor hepatic vein to the proximal inferior vena cava, and donor hepatic artery to the recipient abdominal aorta. The donor biliary tract was anastomosed to the recipient jejunum. In this study, anti-thymocyte globulin (ATG) and anti-CD40mAb were used as immunosuppressive therapy.

The initial postoperative period was characterized by an initial depletion of recipient platelets followed by slight recovery, a stable hematocrit, and persistently low leukocyte levels due to ATG and anti-CD40mAb immunosuppressive therapy. At postoperative day (POD) 15, recipient liver enzymes began to climb, hinting at the first signs of possible graft rejection. In addition, total bilirubin continued to climb throughout the postoperative period, further indicating declining graft function. Graft synthetic function as measured by porcine albumin levels peaked at POD 4 and progressively declined thereafter, consistent with overall worsening graft function. Coagulation parameters, such as PT and aPTT, remained stable throughout the experiment. Despite encouraging laboratory findings, specimens taken at necropsy demonstrated focal hemorrhagic necrosis and thrombotic microangiopathy, consistent with ongoing coagulation dysregulation. Histopathologic specimens taken throughout the postoperative period demonstrated increasing IgG and IgM levels within the graft, consistent with an uncontrolled humoral xenogeneic response. Despite low total lymphocyte levels, analysis of lymphocyte subsets showed a significant increase in the number of CD8^+^ T cells and CD159a^+^ NK cells, consistent with ongoing and/or worsening cell-mediated rejection. In addition, substantial infiltration by recipient macrophages and neutrophils was noted within the graft. Taken together, these findings suggest that an integrated immune response involving both the innate and adaptive immune systems likely continues to exacerbate the consumptive coagulopathy and thrombocytopenia characteristic of species-discordant liver xenotransplantation.

The extensive preclinical experience in pig-to-NHP liver xenotransplantation to date has demonstrated the life-saving potential of liver xenotransplantation. With survival times approaching a month, clinical trials of liver xenotransplantation as a ‘bridge’ to allotransplantation are within reach (23). However, despite decades of work, the definitive combination of genetic modifications and pharmaceutical support required to bring liver xenotransplantation to the clinic continues to elude us due to the two persistent lethal barriers to liver xenotransplantation: 1) rapid, life-threatening thrombocytopenia and 2) uncontrolled consumptive coagulopathy, culminating in lethal hemorrhage.

## Thrombocytopenia and Coagulopathy as Major Barriers to Liver Xenotransplantation

### Thrombocytopenia

In early WT pig-to-NHP models, liver xenografts underwent rapid antibody-mediated rejection, leading to graft destruction and recipient demise within hours (9, 16). The consequences of thrombocytopenia thus took a back seat to the task at hand: overcoming hyperacute rejection. The advent of genetic engineering tools allowed for the establishment of GTKO pigs (24), the organs from which demonstrated drastically reduced hyperacute rejection in pig-to-NHP models (10, 25, 26). In 2010, Ekser et al. (10) demonstrated that severe thrombocytopenia develops within 1 hour of pig-to-NHP orthotopic liver xenotransplantation from GTKO pigs transgenic for a human complement regulatory protein, CD46. The result was spontaneous hemorrhage in multiple organs in the absence of signs of humoral or cellular rejection, ultimately limiting survival times to a maximum of 7 days (10, 27). These observations demonstrated that severe, rapid thrombocytopenia occurred independently of organ rejection, a phenomenon that is not typical for heart or kidney xenotransplantation (13, 18). The conundrum prompted further research into the physiologic and immunologic mechanisms of liver xenotransplantation-induced thrombocytopenia. The current literature suggests severe thrombocytopenia following liver xenotransplantation results from 1) excessive platelet activation/aggregation and 2) aberrant platelet sequestration/phagocytosis.

**Figure 1:**
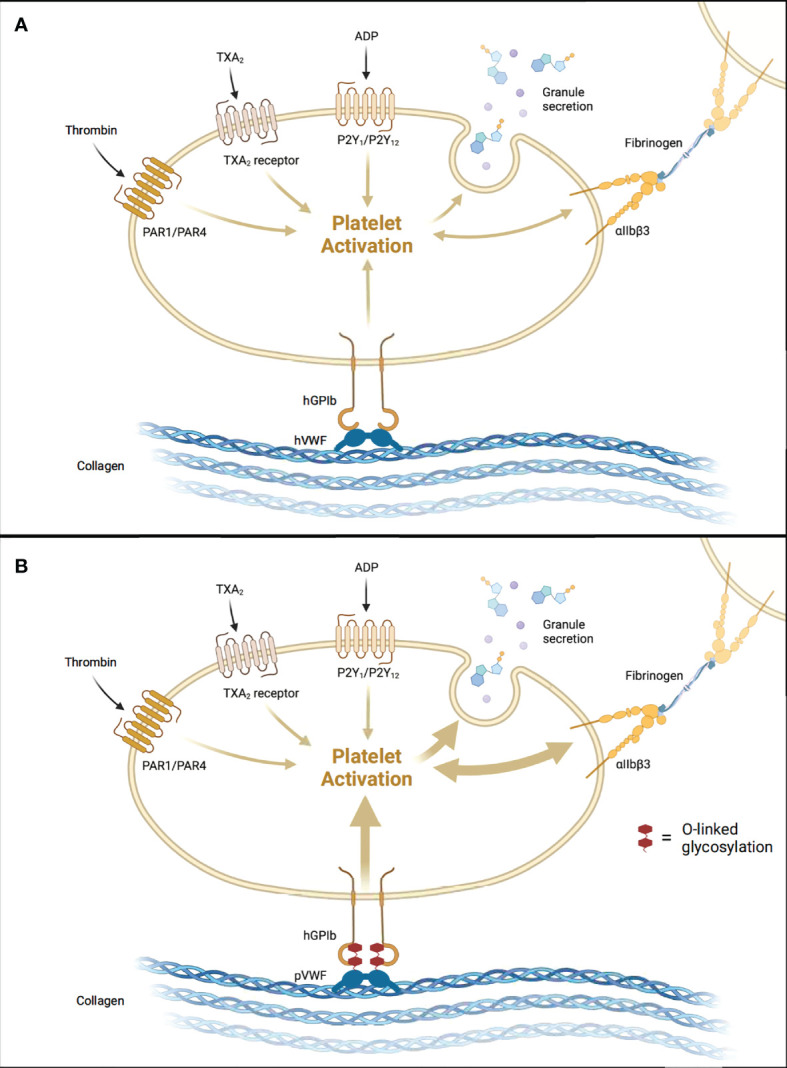
Aberrant Activation of Human Platelets by Porcine vWF. **(A)** Human-to-human allogeneic platelet activation. **(B)** Pig-to-human xenogeneic platelet activation augmented by tighter pvWF-hGpIb binding. hGPIb, human glycoprotein Ib; hVWF, human von Willebrand Factor; pVWF, porcine von Willebrand Factor. Created with BioRender.com.

Platelet activation classically occurs when platelet GpIb receptors bind von Willebrand Factor (vWF) on the exposed negatively charged surfaces of damaged vascular endothelium (**Figure 1**). The GpIb/vWF interaction induces platelet degranulation, releasing fibrinogen, vWF, serotonin, ADP, and Ca^2+^ into the bloodstream. Thromboxane A2 (TXA2) is released by damaged endothelial cells and promotes further platelet degranulation. ADP activates platelets, which causes a structural change that exposes GpIIb/IIIa receptors which then bind fibrinogen, resulting in the formation of a platelet plug. A finite number of platelets are consumed when this process occurs naturally.

In the setting of liver xenotransplantation, there are several points along this pathway that are altered as a result of donor-recipient interspecies incompatibilities. Schulte et al. (28) demonstrated that porcine vWF binds more tightly to human GpIb, resulting in significantly increased platelet activation *in vitro*. This tighter binding was shown to result from increased O-linked glycosylation in porcine vWF compared to human vWF (**Figure 1**) (28). In a subsequent *ex vivo* pig-to-NHP liver xenoperfusion model, human platelet activation, as measured by beta-thromboglobulin levels, was significantly higher in the xenoperfusion groups compared to alloperfusion controls, and the combined addition of anti-GpIb antibodies with DDAVP (desmopressin) slightly reduced platelet activation (not statistically significant) (29). Indeed, all xenoperfusion groups demonstrated significantly lower platelet counts when compared to alloperfusion controls. These findings suggest that porcine vWF/primate GpIb interspecies incompatibilities, specifically increased O-linked glycosylation, increase platelet activation and consumption, and ultimately contribute to liver xenotransplantation-induced thrombocytopenia. Recently, Connolly et al. (30) demonstrated that expression of human vWF in porcine livers significantly reduced platelet consumption in an *ex vivo* liver perfusion model, further supporting this notion.

The increased platelet activation in liver xenotransplantation additionally results in downstream amplification of platelet aggregation. As platelets degranulate, increased vWF and fibrinogen are released and subsequently bound by nearby activated platelets through GpIIb/IIIa receptors. The result is extensive platelet aggregation. This notion is supported by *in vitro* studies demonstrating that baboon platelet aggregation is induced by direct contact with porcine aortic endothelial cells, liver sinusoidal endothelial cells (LSECs), and hepatocytes (31). Baboon platelet aggregation was ameliorated when these porcine cell lines were pre-treated with anti-GpIb and anti-GpIIb/IIIa antibodies (31). Xenoperfusion studies conducted by Burdorf et al. (32) demonstrated anti-GpIb antibodies delayed the development of thrombocytopenia by 2 hours, supporting the notion that dysregulated platelet activation contributes to liver xenotransplantation-induced thrombocytopenia. These findings provide substantial evidence that disrupting components of platelet activation and aggregation reduces platelet consumption which subsequently mitigates the severe thrombocytopenia seen in liver xenotransplantation.

Although there is substantial evidence for increased platelet activation/aggregation in pig-to-NHP liver xenotransplantation models, other studies have also shown that platelet *sequestration* within the xenograft may play a critical role (8, 33). In an *ex vivo* perfusion model of human blood through a wild-type pig liver, 93% of platelets were removed within 15 minutes despite no evidence of endothelial cell or platelet activation (8, 33). Tissue biopsies of the liver xenograft showed extensive platelet phagocytosis by porcine Kupffer cells (KC), and *in vitro* co-culture assays demonstrated human platelet sequestration by porcine LSECs (33). Degraded human platelets were additionally observed inside porcine hepatocytes. These findings provide strong evidence for liver sequestration of recipient platelets by porcine LSECs, Kupffer cells, and hepatocytes as a key mechanism of liver xenotransplantation-induced thrombocytopenia.

### Liver Sinusoidal Endothelial Cells

**Figure 2:**
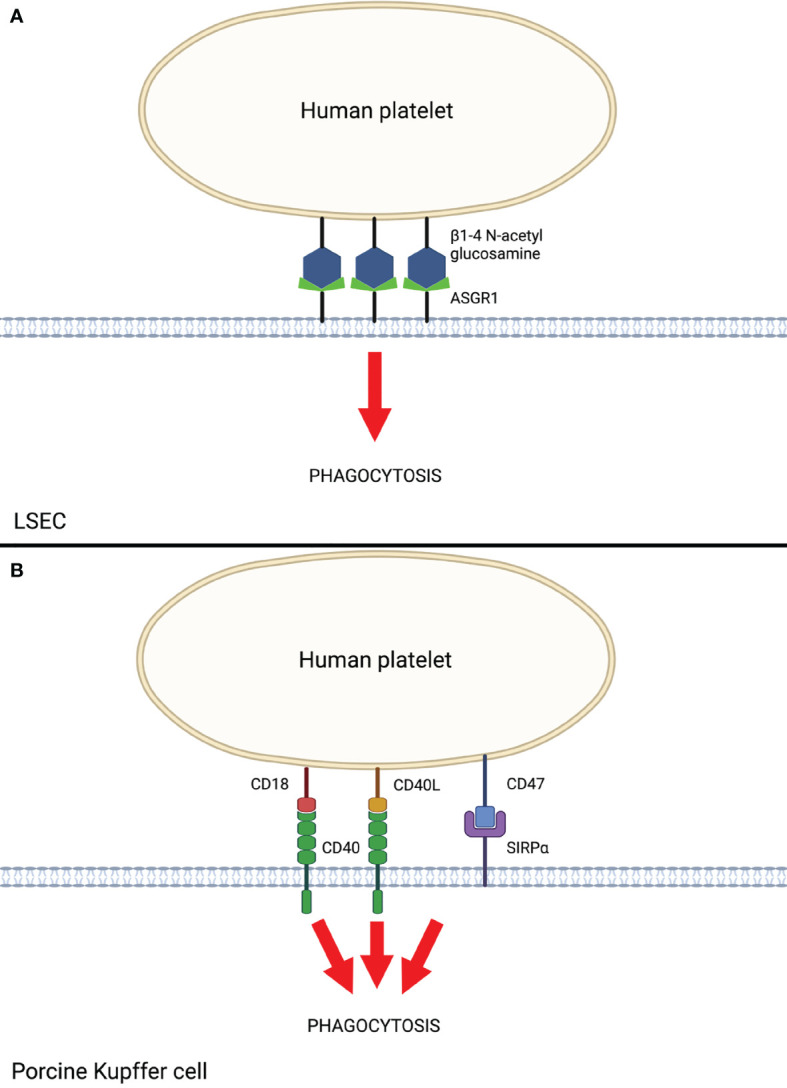
Human Platelet Sequestration by Porcine LSEC and Kupffer Cells. **(A)** Liver sinusoidal endothelial cell (LSEC)-mediated phagocytosis of human platelets *via* ASGR1. **(B)** Porcine Kupffer cell-mediated phagocytosis of human platelets *via* interactions between CD40 and SIRPα with respective ligands on human platelets. Created with BioRender.com.

LSECs play a major role in scavenging bloodborne waste through an intrinsically elevated endocytic capacity and are therefore of particular interest in the setting of species-discordant liver xenotransplantation. Paris et al. (34) demonstrated that porcine LSECs recognize and bind the galactose β1-4 N-acetyl glucosamine (Galb1,4-NacGlc) glycoprotein on human platelets *via* asialoglycoprotein receptor-1 (ASGR1) (**Figure 2**). Elimination of this glycoprotein by treatment with asialofetuin proportionally decreased platelet phagocytosis by LSECs *in vitro* (34). Reduced human platelet phagocytosis in *ex vivo* perfusion through ASGR1-deficient pig livers has also been reported (33). ASGR1-mediated platelet phagocytosis is not specific to porcine LSECs, as it was shown to also occur in porcine aortic and femoral arterial vascular endothelium (11, 35), suggesting ASGR1-mediated platelet phagocytosis may be a generalized mechanism of species-discordant platelet consumption.

### Porcine Kupffer Cells (pKC)

Porcine Kupffer cells (KC) also play a major role in liver xenotransplantation-induced platelet sequestration (9). Chihara et al. (36) demonstrated that CD18 is central to porcine KC recognition of human platelets *in vitro*. Anti-CD18 antibodies and siRNA knockdown of CD18 in pig cells resulted in significantly reduced human platelet binding and phagocytosis (36). The mechanism by which porcine KC CD18 recognizes and phagocytoses human platelets is thought to occur through the recognition of CD40 ligand (CD40L, aka CD154) on activated platelets (37) and β-N-acetyl d glucosamine (β-GlcNac) on cold-activated platelets (13, 36). Porcine KC additionally express CD40, which further augments recognition and phagocytosis of activated human platelets through CD40L. Indeed, monoclonal antibodies directed against the CD40/CD40L complex have demonstrated prolonged liver xenograft survival in pig-to-NHP models when used as part of a costimulation blockade regimen (7, 38). To date, genetic modifications to this axis have not been attempted, likely due to pig viability concerns. Taken together, these findings suggest CD18 and CD40 receptors on porcine KCs are crucial to human platelet recognition and phagocytosis and serve as a primary target for further research to prevent liver xenotransplantation-induced thrombocytopenia.

Macrophages, including porcine KC, predominantly mediate phagocytosis through the interaction of signal regulatory protein *α* (SIRP*α*) on the macrophage with the ubiquitously expressed CD47 on “self” cells, including platelets, red blood cells, and leukocytes. Appropriate species-specific binding of CD47 to SIRP*α* results in dominant inhibitory signaling through immunoreceptor tyrosine inhibitory motifs (ITIMs), leading to the prevention of phagocytosis by the macrophage (39). In the setting of pig-to-human or pig-to-NHP liver xenotransplantation, porcine SIRPα (pSIRPα) exhibits suboptimal binding to human CD47 (hCD47). Porcine KC were shown to phagocytose human platelets in a porcine SIRPα/human CD47-dependent manner *in vitro*, and transgenic expression of human SIRPα on porcine KC significantly reduced human platelet phagocytosis (13). These results provide evidence that genetic modification of organ-source pigs to express human SIRPα may reduce porcine KC-mediated platelet phagocytosis and subsequently mitigate liver xenotransplantation-induced thrombocytopenia.

### Coagulation Dysregulation

Aberrant activation of the coagulation cascade is a hallmark feature of hyperacute rejection. In pig-to-NHP liver xenotransplantation, however, dysregulated coagulation occurs even in the absence of immunologic evidence of hyperacute rejection (13). Interestingly, coagulation dysregulation is a substantially greater barrier to liver xenotransplantation than heart or kidney xenotransplantation (11, 40).

Genetic discordance in tissue factor (TF) and subsequent constitutive activation of the extrinsic pathway is well-supported in the literature as a primary driver for liver xenotransplantation-induced consumptive coagulopathy (41, 42). TF is constitutively expressed in subendothelial fibroblasts and muscle cells and is inducibly expressed in endothelial cells in the setting of systemic inflammation (43). Upon exposure of TF to the bloodstream, it binds and subsequently activates Factor VII, initiating the extrinsic pathway of the coagulation cascade (11, 44). TF activity is regulated by tissue factor pathway inhibitor (TFPI), a polypeptide that binds the TF/VIIa complex and inhibits its activity. Molecular incompatibilities between donor TFPI and recipient TF have been a subject of intensive investigation. Lee et al. (45) initially demonstrated *in vitro* that both recombinant pig TFPI and human TFPI efficiently inhibited the activation of human Factor Xa by human TF/VIIa. More recently, Ji et al. (21) provided *in vitro* evidence that pig TFPI does *not* inhibit human TF as efficiently as human TFPI, suggesting molecular incompatibilities between pig and human may, in fact, contribute to coagulation dysregulation in liver xenotransplantation. Additionally, this group demonstrated that recipient (baboon) TF is activated in pig-to-NHP heterotopic liver xenotransplantation, but donor (pig) TF remains inactivated (21), suggesting the aberrant upregulation of recipient TF combined with genetically incompatible donor TFPI is the key issue. Interestingly, Ahrens et al. (43) recently demonstrated that siRNA knockdown of porcine TF significantly increased clotting time and decreased thrombus formation when compared with WT pigs, suggesting porcine TF contributes to aberrant liver xenotransplantation-induced coagulation dysregulation despite initial evidence to the contrary (21). Preclinical trials involving human TFPI expressing pigs have shown promising results (Kefeng Dou, personal communication); however, the specific contribution of human TFPI as compared to the additional interventions in the trials remains to be clarified. Moreover, it is reported that overexpression of human TFPI on pig cells were compatible with spontaneous bleedings in pigs and eventual death (David Ayares, personal communication).

Species incompatibilities in the thrombin-thrombomodulin complex (TTC) have been proposed as an additional contributing factor to coagulation dysregulation in liver xenotransplantation. Thrombomodulin is an integral membrane protein expressed on the surface of endothelial cells and is a cofactor for thrombin. The interaction of thrombin with thrombomodulin results in the formation of the TTC, which through the activation of protein C inhibits factors Va and VIIIa, resulting in dampening of the coagulation cascade. At the same time, the removal of thrombin inherent to the formation of the TTC further reduces the number of procoagulant factors. As such, appropriate binding of thrombomodulin to thrombin is essential for regulation of the coagulation cascade. *In vitro* studies demonstrate that porcine thrombomodulin binds to human thrombin, but the resulting TTC fails to effectively activate human protein C (46). Transgenic expression of human thrombomodulin (hTM) in porcine aortic endothelial cells resulted in substantially greater activation of human activated protein C *in vitro* (47), providing promising evidence for the utility of hTM transgenic pigs. Organs from hTM transgenic pigs were shown to activate protein C at a substantially higher rate than wild-type pigs; however, this expression was the lowest in the liver (48). Further optimizing hTM expression in porcine livers is necessary to evaluate the true benefit of this genetic modification with respect to liver xenotransplantation, as shown in prolonged survival in preclinical hTM pig-to-NHP kidney (49) and heart (4, 50) xenotransplantation.

## Additional Considerations

### Liver Xenograft Function

Overcoming the current immunologic and hematologic barriers to liver xenotransplantation is crucial, however evaluating whether the transplanted organ will function at a sufficient level to sustain human life is of equal importance. In 2010 the Pittsburgh Group evaluated a comprehensive set of liver xenograft functional parameters in a series of pig-to-baboon orthotopic liver xenotransplants, including porcine albumin, fibrinogen, haptoglobin, plasminogen, almost all coagulation factors, bilirubin, and all liver enzymes (51). Overall hepatic synthetic function was maintained throughout the post-operative period which was documented by the measurement of liver enzymes as well as INR. Although INR levels were acceptable following pig liver xenotransplantation in baboons, many coagulation factors in baboons remained significantly lower than pre-transplant indicating, in most cases, that they were adjusted with the level of porcine coagulation factors (51). Consistent findings were reported by Kim et al. in 2012, most notably the requirement for continuous IV infusion of albumin and the presence of significantly lower porcine coagulation factor levels (19). Taken together, these findings suggest porcine hepatic function was stable after xenotransplantation and closely approximates NHP hepatic function. Although one can argue about the relatively short survival of the recipient baboons in both studies (3 hours to 9 days), a recent study by Shah et al. (7) showed that two baboons survived almost one month with normal liver function following pig liver xenotransplantation. Therefore, there is enough evidence that porcine liver xenografts will provide an adequate function.

### Infectious Disease Transmission

While potential infectious complications are well-characterized in *allo*transplantation and protocols have been developed to minimize transmission, in xenotransplantation, there exists a risk of zoonotic pathogen transmission from the non-human donor (i.e. pig) organ into the immunocompromised human host. In order to address this issue, exclusion lists have been generated to identify pathogenic organisms that could potentially be transmitted from swine to humans through xenotransplantation (52). Transmission of porcine endogenous retroviruses (PERV) has been researched extensively in the transplantation of pig tissues into NHPs, minks, rats, guinea pigs, and severe combined immunodeficiency (SCID) mice, however aside from a single case of transmission in a guinea pig there have been no other documented cases to date (53). Additionally, pig-derived pathogens have not been identified in immunosuppressed humans with the exception of hepatitis E virus (HEV) (2). Since it is essentially impossible to predict which organisms will pose a health hazard until clinical trials are conducted, the current approach to addressing the issue of infectious disease transmission in pig-to-human xenotransplantation is rigorous selection of “pathogen-free” pigs (2). This is achieved through herd isolation, continuous surveillance of source animals, meticulous breeding records, microbiological assessments, and standard veterinary care to ensure the overall health of donor pigs. Breeding swine in biosecure facilities will additionally minimize swine exposure to pathogens. The first genetically modified pig-to-human cardiac xenotransplantation took place on January 7, 2022, a ground-breaking advancement in the field of xenotransplantation. This landmark event will provide a rare and valuable opportunity to observe and evaluate potential infectious complications in a true pig-to-human xenotransplant.

### Patient Consent

There are many ethical considerations and regulatory aspects in xenotransplantation, particularly with respect to patient consent and autonomy. These issues were previously discussed in detail (54, 55). Briefly, in patients with chronic liver failure who retain capacity to make their own medical decisions the issue of patient consent is straightforward. For patients who arrive to the hospital unconscious in fulminant hepatic failure, the current standard is to place them on the waiting list for an emergent human liver allotransplant. The current ethical framework for treating life-threatening emergencies differs substantially from non-emergency situations, in that informed consent is not required in order to treat. In a scenario where the pig liver xenotransplantation is a reasonable alternative, the clinician would be required to choose between placing the patient on the waiting list or proceeding with liver xenotransplantation with an informed consent obtained from the family and/or hospital’s ethical committee.

## Future Directions

Despite the considerable success achieved in preclinical liver xenotransplantation trials with survival of almost one month, there still exists a significant gap between where the field is now and where it needs to be to begin the first clinical pig-to-human liver xenotransplantation trial. There are several potential areas for future research to overcome the major barriers of thrombocytopenia and coagulation dysregulation.

Although pharmaceutical approaches such as anti-GpIb and/or anti-GpIIb/IIIa antibodies have the potential to ameliorate thrombocytopenia, there always exists a risk of unintended adverse events with polypharmacy. Genetic engineering of pigs, therefore, is the preferred approach. Based on preliminary results (30), human vWF has shown potential to reduce platelet consumption. Additional genetic modifications aimed at minimizing platelet sequestration by porcine LSECs and KCs include ASGR1-KO and transgenic expression of human CD18 and/or CD40, respectively. Given the duality of the SIRPα/CD47 interaction, genetic modification of this axis would likely require the concomitant knock-in strategy to delete porcine SIRPα and CD47 and replace them with the human counterparts. This approach may prove technically challenging and threaten pig viability (13), thus further research is needed to evaluate this possibility.

Developing an effective approach to stabilizing coagulation dysregulation in pig-to-NHP liver xenotransplantation models is imperative in order to progress to pig-to-human clinical trials. Pharmaceutical support with coagulation factor replacement and co-stimulation blockade will likely continue to be necessary to achieve sufficient survival times, however, introducing genetic modifications may augment its efficacy. Given the implications of TF in liver xenotransplantation-induced coagulation dysregulation described previously, genetic modifications to porcine TF may prove valuable (43). Additionally, further optimization of transgenic hTM expression in organ source pig livers would provide valuable information regarding its utility. Indeed, novel approaches in genetic engineering of pig cells and testing capabilities of their phenotypes without making those genetically modified pigs each time for each genetic modification will reduce the time and effort to understand which genetic modification combinations would be optimal for preclinical/clinical trials (56, 57). With the strong data in preclinical trials in pig-to-NHP xenotransplantation trials as well as recent progress in ‘first-in-man’ genetically-engineered pig heart xenotransplantation into a patient, we would also strongly suggest that complement regulatory proteins, such as CD55 and CD46 as well as hTM and human EPCR expression would be helpful in extending the xenograft survival (13, 42, 58). Another future gene target amenable to manipulation would be the expression of human albumin in pigs since pig albumin production is significantly low compared to human albumin levels (59). Alterations to baseline physiologic levels of porcine albumin may alter the viability of the donor pig, and could avoid long-term low albumin-related issues (e.g. ascites) in the recipient. Thus, further research is necessary to explore this approach.

## Conclusion

As genetic modification techniques continue to improve, the potential of liver xenotransplantation as a bridge to allotransplantation in fulminant liver failure patients becomes increasingly apparent (23, 60). Since genetically-engineered pigs with multiple gene knockouts and knock-ins have been generated (over ten genes) while maintaining donor viability, at present, there appears to be no fundamental limit to how much the porcine genome can be edited before the well-being of the donor pig is compromised. As such, the genetic modifications proposed here have the potential to greatly improve pig-to-NHP liver xenograft survival. Combined with tailored immunosuppression and coagulation factor support, the first pig-to-human liver xenotransplantation clinical trial may well be just over the horizon.

## Author Contributions

AC-N wrote the first draft under the supervision of BE. All authors contributed to the writing and critical review of the manuscript. All authors contributed to the article and approved the submitted version.

## Funding

Work on xenotransplantation at Indiana University has been supported by internal funds of the Department of Surgery, in part, with support by the Board of Directors of the Indiana University Health Values Fund for Research Award (VFR-457-Ekser), the Indiana Clinical and Translational Sciences Institute, funded in part by Grant # UL1TR001108 from the National Institutes of Health (NIH), National Center for Advancing Translational Sciences, Clinical and Translational Sciences Award, and NIH NIAID R21AI164002.

## Conflict of Interest

The authors declare that the research was conducted in the absence of any commercial or financial relationships that could be construed as a potential conflict of interest.

## Publisher’s Note

All claims expressed in this article are solely those of the authors and do not necessarily represent those of their affiliated organizations, or those of the publisher, the editors and the reviewers. Any product that may be evaluated in this article, or claim that may be made by its manufacturer, is not guaranteed or endorsed by the publisher.

## Abbreviations

ASGR1, asialoglycoprotein receptor-1; ATG, anti-thymocyte globulin; GTKO, alpha galactosyltransferase gene knockout; hPCC, human prothrombin complex concentrate; HXR, hyperacute xenograft rejection; LSEC, liver sinusoidal endothelial cell; mAb, monoclonal antibody; NHP, nonhuman primates; KC, Kupffer cell; SIRPα, signal regulatory protein alpha; TF, tissue factor; TFPI, tissue factor pathway inhibitor; TTC, Thrombin-thrombomodulin complex; vWF, von Willebrand Factor.
